# Baicalin acts as an adjuvant to potentiate the activity of azithromycin against *Staphylococcus saprophyticus* biofilm: an in vitro, in vivo, and molecular study

**DOI:** 10.1186/s13567-022-01088-z

**Published:** 2022-10-12

**Authors:** Jinli Wang, Siya Li, Jinwu Meng, Jinyue Zhu, Tianxin Qiu, Wenjia Wang, Jinxue Ding, Zhenguang Liu, Kun Li, Mujahid lqbal, Deyun Wang, Yi Wu, Jiaguo Liu

**Affiliations:** 1 MOE Joint International Research Laboratory of Animal Health and Food Safety and Traditional Chinese Veterinary Medicine Research Center, College of Veterinary Medicine, Nanjing Agricultural University, Nanjing, 210095 China; 2 Department of Pathology, Cholistan University of Veterinary and Animal Sciences (CUVAS), Bahawalpur, 63100 Pakistan

**Keywords:** *S. saprophyticus*, baicalin, azithromycin, biofilm, WalK/R system

## Abstract

*Staphylococcus saprophyticus* is frequently involved in various difficult-to-treat infections due to the formation of biofilms. To identify useful antibiofilm strategies, this study explored the efficacy and mechanism of baicalin in enhancing the ability of azithromycin against multidrug-resistant *Staphylococcus saprophyticus*-Liu-2016-Liyang, China-francolin (MDRSS) biofilms in vitro and in vivo. When azithromycin was used in combination with baicalin, the minimum inhibitory concentration in biofilm (MICB) for azithromycin decreased 4- to 512-fold. Compared with the azithromycin and baicalin groups, the combination of azithromycin and baicalin could not reduce the biofilm biomass, but the dispersion rates of biofilm were decreased and the bactericidal ability was increased. Furthermore, the relative transcript levels of WalK/R system-related genes were upregulated by the addition of baicalin or azithromycin plus baicalin compared with that of the azithromycin and blank control groups. The strong correlation relationship between the WalK/R system and the bactericidal index demonstrated that baicalin enhanced the bactericidal effect of azithromycin on MDRSS biofilms by modulating the WalK/R system. In the mouse cutaneous infection model, the combination of azithromycin and baicalin succeeded in eradicating MDRSS and decreasing pathological injuries. This study indicated that baicalin has the potential to be an adjuvant to enhance the antimicrobial activity of azithromycin against MDRSS in the biofilm form by modulating the WalK/R system.

## Introduction

The opportunistic pathogen *Staphylococcus saprophyticus* is a gram-positive coccus that has emerged as an important aetiologic agent of bovine mastitis [1], urinary tract infections [2], and francolin ophthalmia [3]. Importantly, staphylococci form biofilms and then enter a metabolically inactive and antibiotic-tolerant persister state to evade antibiotic stress [4]. Additionally, the capacity of staphylococci to form biofilms is a virulence factor that facilitate colonization and adhesion to the epithelium, resulting in recurrent or persistent infections [5, 6]. It is estimated that approximately 80% of chronic infections are associated with biofilms [7]. Once bacteria form a mature biofilm, maximum resistance to antibiotics is achieved [8]. For some antibiotics, eradicating sessile bacteria requires a thousand times greater concentrations than that necessary to inactivate the same strain in its planktonic form [9]. Azithromycin is a macrolide antibiotic that is widely used to treat staphylococcal and streptococcal infections in poultry [10]. It has been proposed that 60% of coagulase-negative staphylococci (CNS) isolated from various samples are highly resistant to penicillin (90%), cotrimoxazole (60%), azithromycin (60%), and ceftriaxone (40%) [11]. However, the available options for treating bacterial infections are reducing due to the rapid dissemination of multidrug-resistant (MDR) pathogens [12]. Therefore, alternative and innovative treatment strategies are urgently needed to overcome such problems.

Many investigations have indicated that diverse antibiotic adjuvants enhance the potency of antibiotics and delay the development of resistance [13]. We previously revealed that the combination of azithromycin and baicalin exerted synergistic effects against multidrug-resistant *Staphylococcus saprophyticus*-Liu-2016-Liyang, China-francolin (MDRSS) [3]. Baicalin, a natural flavonoid compound extracted from *Scutellaria baicalensis Georgi*, exerts remarkable synergistic antistaphylococcal activity with antibiotics [14]. However, the response of azithromycin and baicalin against MDRSS in the biofilm form has not been evaluated.

The membrane and wall of bacterial cells are the targets of numerous traditional Chinese medicines [15]. In a study performed by Zhang et al., it was reported that the cell membrane was damaged after a treatment using baicalin in methicillin-resistant *Staphylococcus aureus* (MRSA) [16]. In staphylococci, the WalK/R (also known as YycG/YycF) two-component system (TCS) plays a significant role in cell wall metabolism. The WalK/R system is highly conserved and specific to gram-positive bacteria with a low G + C content. Peptidoglycan biosynthesis and degradation are positively controlled by the WalK/R system [17]. In staphylococci, most cell wall-anchored proteins, which are components of the MDRSS biofilm matrix [18], have a common cell wall-targeting motif (LPXTG) and are attached to peptidoglycan in the cell wall via sortase A (SrtA) [19].

Taken together, we hypothesized that baicalin promotes the metabolism of peptidoglycan and decreases the anchoring of MSCRAMMs by modulating the WalK/R system. Then, the permeability of azithromycin was increased and acted synergistically with baicalin. Until now, the effect of baicalin as an adjuvant to enhance the antibiofilm properties of azithromycin has not been clearly understood. This study evaluated the role of baicalin in improving the bactericidal activity of azithromycin against sessile bacteria embedded in biofilms by modulating the WalK/R system. Additionally, the effect of azithromycin and baicalin on limiting MDRSS biofilm infection in mice was investigated.

## Materials and methods

### Microbial strains, medium and culture conditions

The MDRSS (azithromycin MIC of 1000 mg/L) used in this study was collected from domestic *Francolinus pintadeanus* with ophthalmia from Jiangsu Province, China. In our previous study, the name of MDRSS was named ARSS [3, 18, 20]. The strain was cultured at 37 °C on Mueller–Hinton Broth (MHB) (Hopebio, Qingdao, China) or Brain Heart Infusion Broth (BHIB) (Solarbio, Beijing, China).

### Susceptibility testing of biofilm

To detect the antibiofilm activity of baicalin and azithromycin, the minimum inhibitory concentrations of the drugs for biofilm cells (MICB) were determined by the two-fold serial dilution method. Biofilms were prepared as described previously [21] with some modifications. Briefly, overnight MDRSS cultures were diluted to 1 × 10^5^ CFU/mL in BHIB. Bacteria were dispensed in 100 μL into each well of a 96-well polystyrene flat-bottom microtiter plate. To promote biofilm formation, the plates were statically incubated for 24 h at 37 °C in a moist environment. After incubation, the medium was discarded, and the biofilms were washed gently with phosphate-buffered saline (PBS) to remove the nonadherent bacteria. Baicalin and azithromycin solutions were serially diluted (two-fold) in MHB to obtain concentrations ranging from 18 000 to 562.5 mg/L and 24 000 to 11.72 mg/L, respectively. Then, the diluted solutions were aerobically coincubated with preformed biofilm at 37 °C for 18 h. The MICB was recorded as the lowest concentration of the drug that inhibited visible growth [22].

### Checkerboard assays on preformed biofilms

To measure the adjuvant capacity of baicalin on the antibiofilm activity of azithromycin, a checkerboard assay was carried out. Mature MDRSS biofilms were prepared as mentioned above in the subsection “Susceptibility testing of biofilms”. Azithromycin and baicalin were serially diluted (two-fold) across the columns and rows of a 96-well plate, and 100 μL of these mixtures was added to the biofilms. The concentration ranges of azithromycin and baicalin were the same as those used for susceptibility testing of biofilms. The plate was incubated for 24 h at 37 °C without agitation. The MICB was defined as the lowest concentration of the drug with no visible growth. The fractional bactericidal concentration index (FBCI) was used to determine the synergistic or antagonistic interaction of drugs. The FBCI of each effective combination (i.e., all of the wells corresponding to a MICB) in the microtitre plate was calculated with the following formula:

$$\mathrm{FBCI}=\frac{MICB \ of \ azithromycin \ in \ combination}{MICB \ of \ azithromycin \ alone}+\frac{MICB \ of \ baicalin \ in \ combination}{MICB \ of \ baicalin \ alone}$$

An FBCI value > 4 indicates antagonism, 0.5–4 indicates no interaction, and ≤ 0.5 indicates synergy [23].

## The eradication effect of azithromycin and baicalin on preformed biofilms

To detect the eradication effect of azithromycin and baicalin on preformed biofilms, the biofilm biomass was measured after the preformed biofilm was exposed to drugs for 24 h. Briefly, mature biofilms were prepared according to the method described above in the subsection “Susceptibility testing of biofilms”. The biofilms were then incubated for another 24 h at 37 °C in fresh MHB (blank control group), MHB with 1/4 MICB baicalin (Bac group), MHB with 1/32 MICB azithromycin (Azm group), and 1/32 MICB azithromycin plus 1/4 MICB baicalin (Azm + Bac group). After incubation, the biomass of biofilms in each well was evaluated by the crystal violet staining method. Each well was washed carefully with sterile PBS to remove the planktonic cells, fixed with 2.5% glutaraldehyde for 1.5 h, and finally air-dried. The biofilm in the wells was stained using 1% (wt/vol) crystal violet for 20 min. Then, the wells were thoroughly rinsed with PBS until the wells in the negative control (without biofilms) became colourless. Ultimately, 33% glacial acetic acid was added to dissolve the absorbed crystal violet. Absorbance at 570 nm (*A*_*570*_) was detected by a Multiskan FC microplate reader (Thermo, USA).

### Biofilm dispersion assay

To investigate the influence of drugs on biofilm dispersion, a biofilm dispersion assay was conducted as previously described with slight modifications [24]. Mature MDRSS biofilms were prepared in a 96-well polystyrene flat-bottom microtiter plate as described above in “Susceptibility testing of biofilm” section. The following groups based on different treatments were formed: Bac, Azm, Azm + Bac, and blank control groups. Bacteria were challenged with baicalin (Bac group), azithromycin (Azm group), and azithromycin plus baicalin (Azm + Bac group). The blank control group was treated with an equal volume medium. The final concentrations of azithromycin and baicalin were 1/32 MICB and 1/4 MICB, respectively. After incubation, the bacteria in the supernatant (C_s_) and bottom (C_b_) of wells were collected and serially diluted (1:10) in PBS to determine the colony-forming units (CFU). The ratios of dispersion were calculated with the formula C_s_/(C_s_ + C_b_). Each experiment was conducted for three independent biological replicates.

### The bactericidal effect of azithromycin and baicalin

To detect the bactericidal effect of azithromycin and baicalin, the CFU counts and the ratio of PI/total fluorescence intensity were measured.

For the quantification of cultivable cells, the mature biofilms were prepared and treated as mentioned above in “The eradication effect of azithromycin and baicalin on preformed biofilms” section. Then, the formed biofilm was dispersed by sonication for 20 min, and the bacterial supernatant was collected, serially diluted 1:10 in PBS and plated on mannitol salt agar (MSA) plates. After incubation for 18–24 h at 37 °C, the colonies were counted, and the number of living cells was reported as log_10_ CFU.

For the ratio of PI/total fluorescence assay, one millilitre of 1 × 10^5^ CFU/mL MDRSS cultures was added to a 6-well plate containing a cover slide to grow biofilms. After 24 h, the biofilms were carefully washed with PBS. Then, azithromycin and baicalin separately and in combination were added and cultured for 24 h. The final concentrations of azithromycin and baicalin were 1/32 MICB and 1/4 MICB, respectively. After incubation, the medium was discarded, and the fluorescent LIVE/DEAD Baclight™ bacterial viability kit L7012 (Molecular Probes, Invitrogen) was used to stain the adherent organisms according to the instructions from the manufacturer. In each sample, SYTO9 and PI (1 mL final volume) were added and incubated in the dark for 15 min at room temperature. Eventually, these slices were washed thoroughly with 0.85% NaCl. Fluorescent images were observed under an A1 confocal laser scanning microscope (CLSM) (Nikon, Japan).

### Analysis of MDRSS morphology by scanning electron microscopy

The influence of drugs on MDRSS morphology was examined by scanning electron microscopy (SEM). Biofilms were prepared in a 24-well polystyrene flat-bottom microtiter plate containing an 8 mm round cover slide as described above in “The bactericidal effect of azithromycin and baicalin” section. The biofilms were coincubated with baicalin, azithromycin, and the combination of azithromycin and baicalin. The incubation was performed at 37 °C for 24 h. The final concentrations of azithromycin and baicalin were 1/32 MICB and 1/4 MICB, respectively. After incubation, each biofilm slice was rinsed with PBS and fixed in 2.5% glutaraldehyde overnight at 4 °C. Later, the slices were dehydrated using ethanol gradients, dried, and coated with gold for SEM observation (S3400N, Hitachi, Japan).

### Alkaline phosphatase and β-galactosidase contents in the biofilm supernatant

To quantify the cellular permeability induced by anti-MDRSS treatments, mature MDRSS biofilms were prepared in a 24-well plate and then cultured with azithromycin, baicalin or azithromycin plus baicalin as mentioned above in “The eradication effect of azithromycin and baicalin on preformed biofilms” section. After incubation for 24 h, the supernatants were collected and filtered through 0.22 μm syringe filters. Then, the contents of alkaline phosphatase (AKP) and activities of β-galactosidase (β-GAL) were determined using AKP assay kits (Solarbio, Beijing, China) and β-GAL assay kits (Solarbio, Beijing, China) following the instructions from the manufacturer.

### The transcript levels of WalK/R system-associated genes detected by RT‒PCR

To determine the influence of drugs on the WalK/R system, the transcript levels of WalK/R system-associated genes (*WalK*, *WalR*, *yycI*, *yycH*) in MDRSS biofilms were assessed using the RT‒PCR method. The primers were designed by Primer 6.0 software, and the sequences are listed in Table 1. The expression levels of the housekeeping gene 16S rRNA were used to normalize the expression of target genes. The biofilms were prepared and handled as mentioned above in “The eradication effect of azithromycin and baicalin on preformed biofilms” section. The biofilms from each group were collected for RNA extraction. RNAiso Plus Reagent (Angle, China) was used to extract total RNA. The concentration of RNA was measured using a NanoDrop™ One (Thermo, USA). cDNA was generated from the extracted RNA using a Hiscript II 1st Strand cDNA Synthesis Kit (Vazyme, China) according to the manufacturer’s instructions. Real-time PCR was performed with ChamQ SYBR qPCR Master Mix (Vazyme, China) on a StepOne PCR instrument (Applied Biosystems, USA) in compliance with the manufacturer’s instructions. The cycling protocols were performed as follows: holding stage, 95 °C for 3 min; cycling stage, 95 °C for 10 s and 60 °C for 60 s, 40 cycles; melt curve stage, 95 °C for 15 s, 60 °C for 60 s, and 95 °C for 15 s.

**Table 1:** **Sequences of primers used in this study**

Target gene	Primer	Sequence (5′–3′)	Accession number	Amplicon size (bp)	Gene region
*16S rRNA*	*16S rRNA*-F	AGTTGTTCTCAGTTCGGATT	NZ-CP031196.1	228	786,530–786,739
	*16S rRNA*-R	ATACGGCTACCTTGTTACG			
*WalK*	*WalK*-F	TTGATTACCGTGATACTTGG	NZ-CP031196.1	195	28,795–28,972
	*WalK*-R	ATTCGCTTGTGCTTCTTG			
*WalR*	*WalR*-F	GTATGGAAGTATGTCGTGAAG	NZ-CP031196.1	197	27,683–27,861
	*WalR*-R	TTGTGCTGGTTGTGAGTAA			
*yycI*	*yycI*-F	AGAAGGTGCGAATAACGATA	NZ-CP031196.1	127	31,912–32,019
	*yycI*-R	AACGGTATAGTAGCCAAGTC			
*yycH*	*yycH*-F	GCCATTAGCAACCTACTTAG	NZ-CP031196.1	262	30,421–30,663
	*yycH*-R	TGTCGCTCTATCAATCGTAT			

### Mouse cutaneous abscess infection model

To evaluate the adjuvant capacity of baicalin on the antibiofilm activity of azithromycin in vivo, the cutaneous abscess infection model in mice was established with some modification as previously described [25]. A total of 50 mice (that weighed 18–22 g) were allocated randomly into the following groups: the baicalin (Bac)-treated group, azithromycin (Azm)-treated group, azithromycin and baicalin combination (Azm + Bac)-treated group, MDRSS (SS)-treated control group, and blank control (BC)-treated group. Cyclophosphamide was injected intraperitoneally at a dosage of 30 mg/kg/day from Day 1 to Day 3. On the 3rd day, sterile 1 cm tube segments were subcutaneously implanted on both flanks. On the 4th day, all mice except those in the BC group were injected with 2 × 10^8^ CFU MDRSS suspensions surrounding the implants. Treatment was initiated on the 7^th^ day, once a day for 3 consecutive days. In the Bac group, mice were injected subcutaneously with baicalin at a dosage of 30 mg/kg. In the Azm group, the mice were treated with 75 mg/kg azithromycin, and in the Azm + Bac group, the mice were treated with azithromycin (75 mg/kg) and baicalin (30 mg/kg); the others were administered an equal volume of normal saline.

The mice were euthanized by cervical dislocation at 24 h after the last treatment. To assess the colonization of bacteria, the implants and the surrounding tissues were collected, weighed, homogenized, serially diluted, and plated on MSA plates containing 0.488 mg/L azithromycin to enumerate the CFU. For histopathological examination, the surrounding tissues were fixed overnight in 4% paraformaldehyde and then embedded in paraffin. Sections (4 μm thickness) were cut and stained with haematoxylin and eosin (H&E) and then examined under light microscopy. Blood samples were also obtained from eyeballs for routine blood examination using the BC-2800 Automated Haematology analyser (Mindray, China). Finally, the remaining tissues were flash-frozen in liquid nitrogen and stored. The concentrations of IL-1β, TNF-α, CXCL_2_, and CCL_2_ in the tissue were measured by ELISA kits (Angle, China) in compliance with the instructions from the manufacturer.

### Data analysis

Correlations among the transcript levels of WalK/R system-associated genes and markers of bactericidal effect were performed using Spearman’s correlation coefficient. The relative gene expression data were determined using the 2^−ΔΔCT^ method. The statistical analysis of other data was analysed using the Kruskal‒Wallis test followed by the post hoc Wilcoxon-Mann‒Whitney or Dunn test by the SPSS Software Package Version 20.0 (IBM, USA) among different groups. The results are presented as the mean ± standard deviation (SD). For all the analyses, a *p* value to or less than 0.05 was considered significant.

## Results

### Baicalin synergizes with azithromycin against MDRSS biofilms

To determine the synergistic efficacy of baicalin-azithromycin combinations against the biofilm cells of MDRSS, baicalin was combined with azithromycin and tested against preformed biofilms of MDRSS. The results are presented in Table 2. When used alone, the MICBs of azithromycin and baicalin against biofilm cells of MDRSS were 6000 and 9000 mg/L, respectively. However, a strong enhancement in antimicrobial activity was found when the combination of azithromycin and baicalin was used (Table 3). During the administration of azithromycin and baicalin, the MICBs of azithromycin decreased 4- to 512-fold. The FBCI values for baicalin with azithromycin ranged from 0.25 to 0.5020. These results suggest that azithromycin and baicalin have a synergistic effect.

**Table 2:** **The MICB concentrations of azithromycin and baicalin against MDRSS biofilms (mg/L)**

	Baicalin	Azithromycin
MICB	9000	6000

MICB: minimum inhibitory concentration of the drugs for biofilm cells (MICB).

**Table 3:** **Combined activity of azithromycin with baicalin against MDRSS biofilm**

Concentration of baicalin (mg/L)	MICB of azithromycin (mg/L)	Recovery fold	FBCI
0	6000	N.A	N.A
4500 (1/2 MICB)	11.72	512	0.5020
2250 (1/4 MICB)	187.5	32	0.2813
1125 (1/8 MICB)	750	8	0.25
562.5 (1/16 MICB)	1500	4	0.3125

MICB: minimum inhibitory concentration of the drugs for biofilm cells (MICB), FBCI: fractional bactericidal concentration index, N.A: not applicable.

### The combination of azithromycin and baicalin did not eradicate mature biofilm but inhibited biofilm detachment

**Figure 1:**
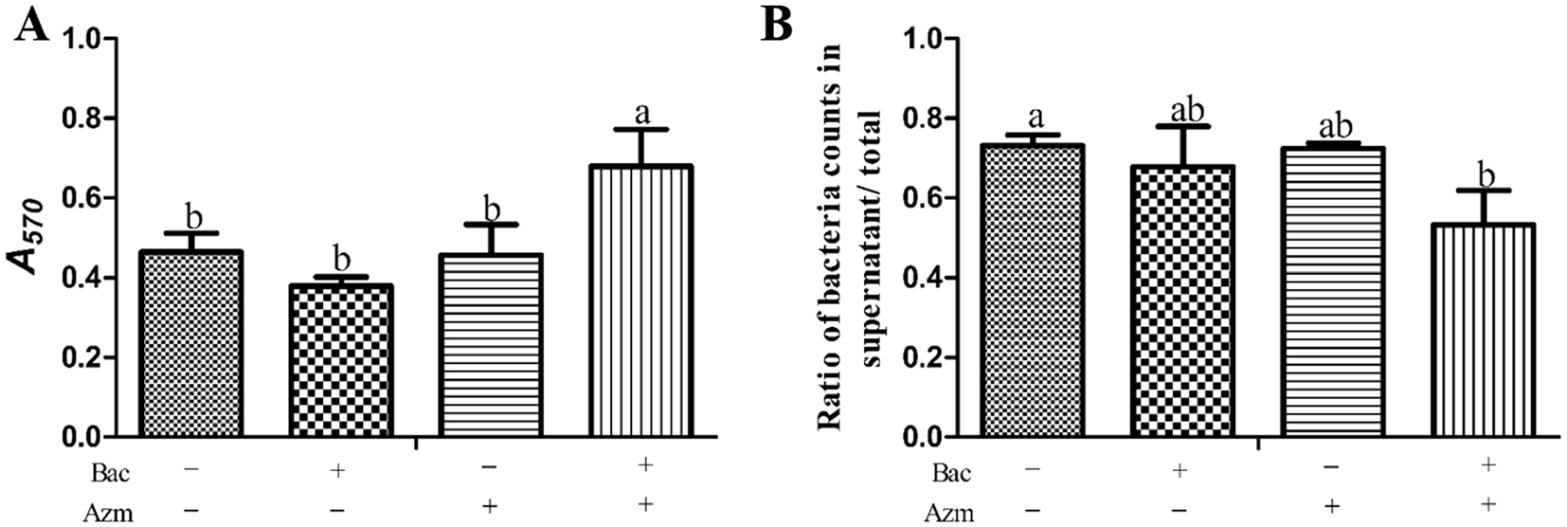
**The combination of azithromycin and baicalin did not eradicate mature biofilms but inhibited detachment.** Bac: baicalin; Azm: azithromycin. **A** Graph shows the means of the *A*_*570nm*_ ± SD obtained by crystal violet staining assays. **B** Dispersion assay. The bacterial counts in the supernatant and bottom were determined by a ten-fold. serial dilution method. The ratios of bacterial counts in supernatant/total were used to represent the ratio of dispersion. All the values were obtained in triplicate in three independent experiments. In the same index, graph bars in a–b with different letters on top represent statistically significant results (*p* < 0.05), whereas bars labelled with the same letter correspond to results with no statistically significant differences.

To investigate the eradication and detachment effects of azithromycin and baicalin on preformed biofilms, the biofilm mass and rates of dispersion were detected. The biofilm mass of MDRSS was not reduced in the presence of baicalin or azithromycin (Figure 1A). After the addition of azithromycin and baicalin, the biofilm mass was significantly higher than that in the other groups (*p* < 0.05). In the dispersion assay, the combination of azithromycin and baicalin markedly inhibited the dispersion of the preexisting biofilm of MDRSS compared with that of the untreated groups. There was no significant difference among the Bac, Azm, and blank control groups. However, the rate of dispersion in the Bac group was lower than that in the Azm and blank control groups (Figure 1B). These results reveal that baicalin can not enhance the effect of azithromycin in eradicating biofilms but inhibits biofilm detachment.

### Baicalin enhanced the bactericidal effect of azithromycin on preformed biofilms

**Figure 2:**
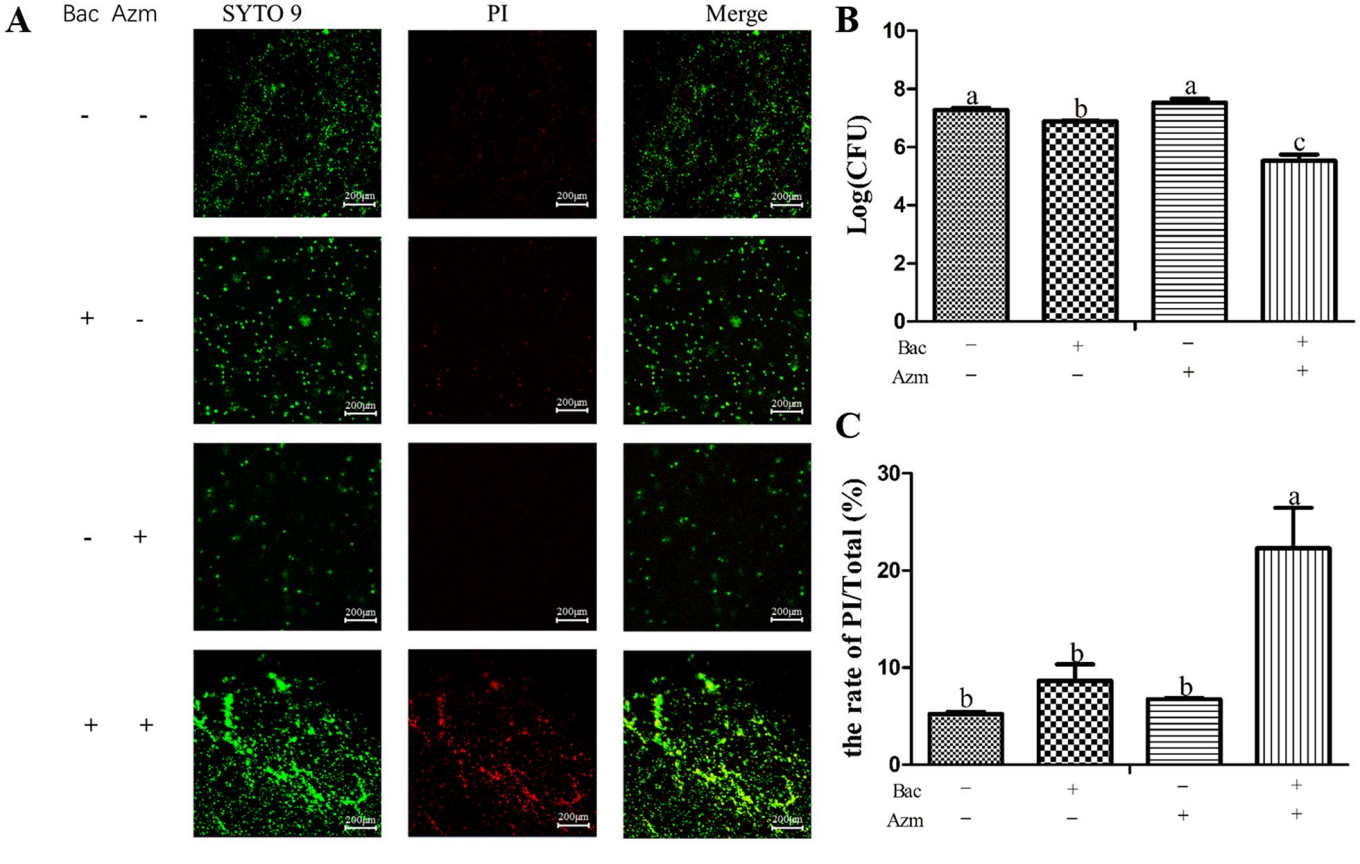
**Baicalin enhanced the bactericidal effect of azithromycin on preformed biofilms.** Bac: baicalin; Azm: azithromycin. **A** Confocal laser scanning microscopy images of azithromycin- and baicalin-treated preformed MDRSS biofilms. The biofilms were stained with a Live/Dead BacLight™ kit. Live and dead cells were stained green and red, respectively. Yellow indicates the superposition of green and red. White scale bars = 200 μm. The **B** graph represents the means of the log CFU ± SD obtained by viable count. **C** The ratio of PI fluorescence intensity/total fluorescence intensity. All the values were obtained in triplicate in three independent experiments. In the same index, the graph bars in a–c with different letters on top represent statistically significant results (*p* < 0.05), whereas the bars labelled with the same letter correspond to results with no statistically significant differences (*p* > 0.05).

To investigate whether baicalin could enhance the bactericidal effect of azithromycin on MDRSS biofilm, the quantifications of cultivable cells were detected after the MDRSS biofilm was exposed to azithromycin and baicalin. As shown in Figure 2B, the combination of azithromycin and baicalin significantly reduced the quantification of cultivable cells, with a 56-fold decrease compared to that of the Bac, Azm, and blank control groups (*p* < 0.05). The quantifications of cultivable cells in the Bac group were remarkably lower than those of the blank control and Azm groups (*p* < 0.05). Then, these results were verified by CLSM. In the Azm + Bac group, most of the bacteria were stained with PI. However, only a few bacteria were stained with PI in the other groups (Figure 2A). The combination of azithromycin and baicalin significantly increased the rates of PI fluorescence intensity/total fluorescence intensity compared with that of the other groups (*p* < 0.05, Figure 2C). No significant difference was observed in the rates of PI/total fluorescence intensity among the blank control, Bac, and Azm groups (*p* > 0.05). However, the rate of PI/total fluorescence intensity in the Bac group was higher than that in the Azm and blank control groups.

### Morphological changes following azithromycin and baicalin

**Figure 3:**
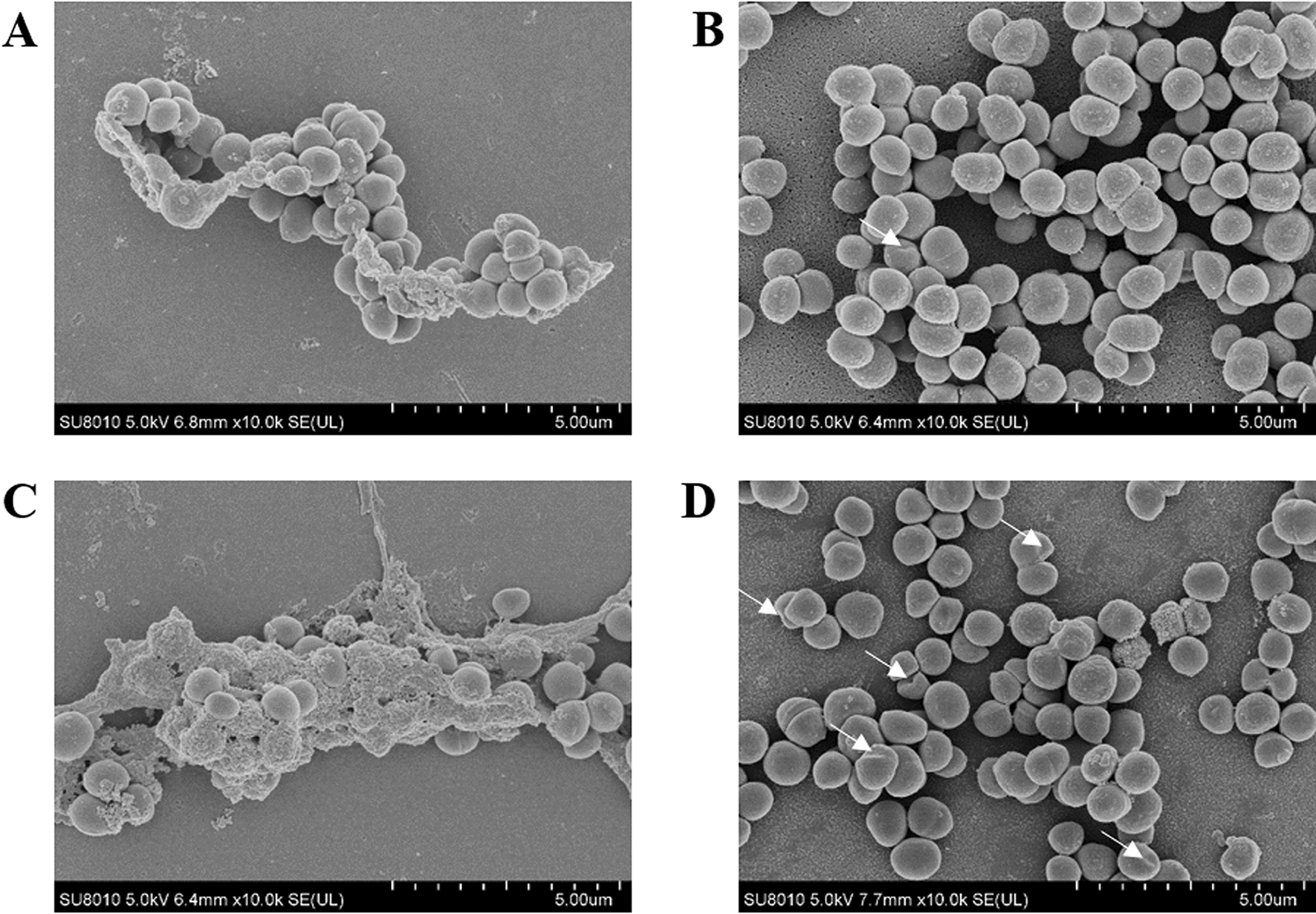
**Morphological changes following azithromycin and baicalin treatment were analysed by SEM.** The white arrows indicate irregular cell wall changes. **A** Blank control group; **B** baicalin (Bac) group; **C** azithromycin (Azm) group; **D** the combination of azithromycin and baicalin (Azm + Bac) group.

In the SEM study, the cells in the blank control and Azm groups appeared intact, plump, and typically sphere-shaped with a smooth exterior, and the bacteria were enwrapped with extracellular polymeric substances (EPS) (Figure 3A and C). Few bacteria displayed an irregular and rugged surface. These morphological variations induced by the combination of azithromycin and baicalin treatment are shown in Figure 3D, which shows that the combination of azithromycin and baicalin could result in damage to bacterial cells. In the Bac group, a few cells were damaged (Figure 3B). Simultaneously, in the Bac and Azm + Bac groups, the surface of bacteria was smooth without EPS. These results were identical to the CLSM and antibacterial assays.

### Baicalin potentiated the efficacy of azithromycin on bacterial permeability

**Figure 4:**
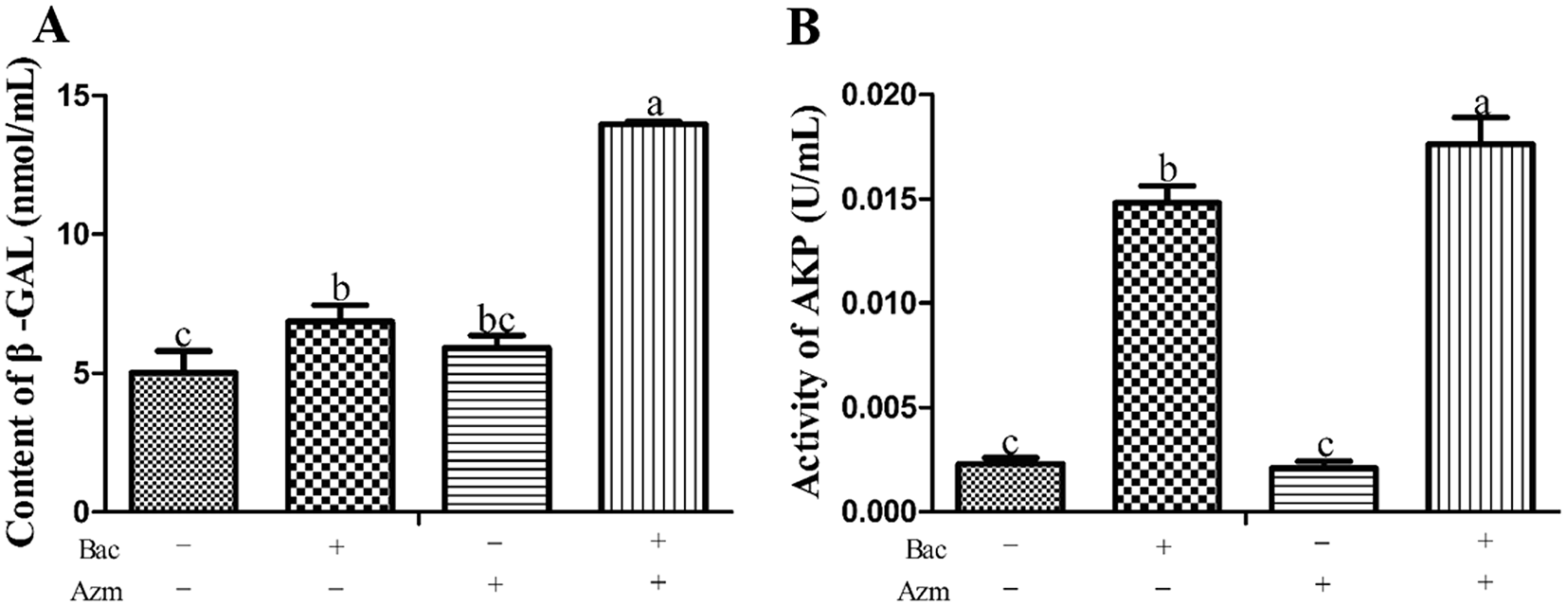
**The extracellular contents of β-GAL and AKP activities.** Bac: baicalin; Azm: azithromycin. **A** The contents of β-GAL in supernatants. **B** AKP activity in the supernatant. In the same index, graph bars in a–c with different letters on top represent statistically significant results (*p* < 0.05), whereas the bars labelled with the same letter correspond to results with no statistically significant differences.

To detect the influence of azithromycin and baicalin on bacterial permeability, the release of AKP and β-GAL into the supernatants was measured. As seen in Figure 4, after baicalin plus azithromycin was added to the wells containing preformed biofilms, the activities of AKP and the contents of β-GAL in the supernatants were significantly increased compared to those of the other groups (*p* < 0.05). The AKP activities in the Bac group were remarkably higher than those in the Azm and blank control groups (*p* < 0.05). Regarding the contents of β-GAL, the Bac group was only significantly higher than that of the blank control group (*p* < 0.05). There was no pronounced difference between the blank control and Azm groups (*p* > 0.05). These results suggest that baicalin can improve the efficacy of azithromycin in destroying the cell walls and membranes of bacteria, which finally leads to the leakage of AKP and β-GAL.

### Effects of azithromycin and baicalin on the transcript levels of WalK/R system-associated genes

**Figure 5:**
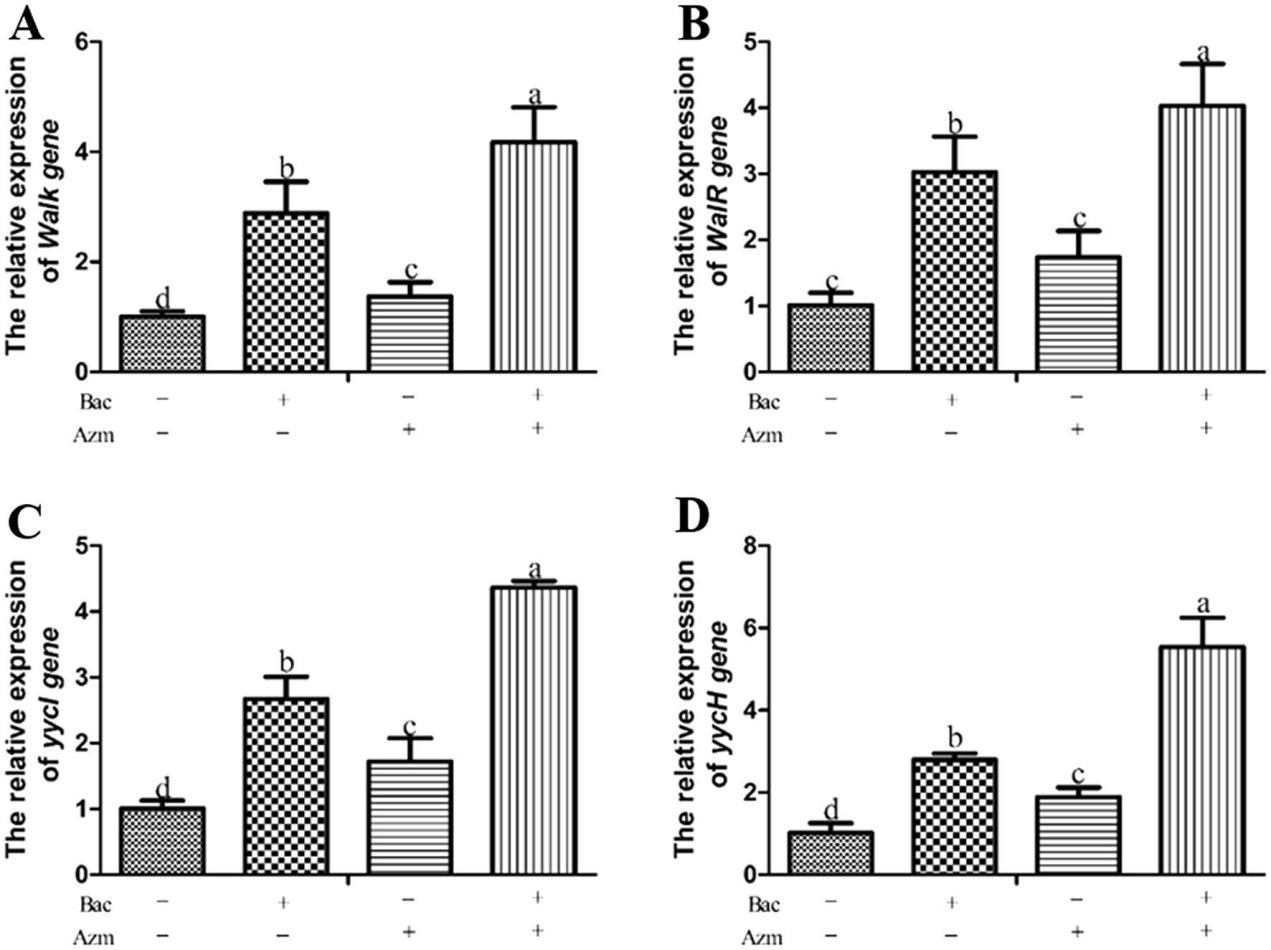
**Effects of azithromycin and baicalin on the transcript levels of WalK/R system-associated genes.** Bac: baicalin; Azm: azithromycin. The transcript levels of the *WalK* (**A**), *WalR* (**B**), *yycI* (**C**), and *yycH* (**D**) genes were detected by RT-PCR. In the same index, graph bars in a–d with different letters on top represent statistically significant results (*p* < 0.05), whereas bars labelled with the same letter correspond to results with no statistically significant differences.

The transcript levels of WalK/R system-associated genes were measured by RT‒PCR to determine the effects of azithromycin and baicalin on the WalK/R system. As illustrated in Figure 5, compared with the Azm and blank control groups, baicalin alone markedly increased the transcript levels of WalK/R system-associated genes (*p* < 0.05). The transcript levels of WalK/R system-associated genes in the Azm + Bac group were significantly higher than those in the other groups (*p* < 0.05).

### Spearman’s correlation coefficients between the WalK/R system and bactericidal index

The correlation coefficients among the WalK/R system and bactericidal index are shown in Table 4. These results revealed that the transcript levels of WalK/R system-associated genes were positively correlated with the contents of β-GAL and the activities of AKP in supernatants. An extremely high positive correlation was observed between the transcript levels of WalK/R system-associated genes and the contents of β-GAL (*p* < 0.01).

**Table 4:** **Spearman’s correlation coefficients between the WalKR system and the bactericidal index**

	WalK	WalR	yycI	yycH	β-GAL	AKP
WalK	1	1.000^**^	1.000^**^	1.000^**^	1.000^**^	0.800
WalR		1	1.000^**^	1.000^**^	1.000^**^	0.800
yycI			1	1.000^**^	1.000^**^	0.800
yycH				1	1.000^*^^*^	0.800
β-GAL					1	0.800
AKP						1

^**^p < 0.01. 0.9–1.0 perfect correlation, 0.7–0.9 strong correlation, 0.4–0.6 moderate correlation, 0.1–0.3 weak correlation, 0.0–0.1 no correlation.

### Baicalin strengthened the efficacy of azithromycin in the treatment of biofilm infection

**Figure 6:**
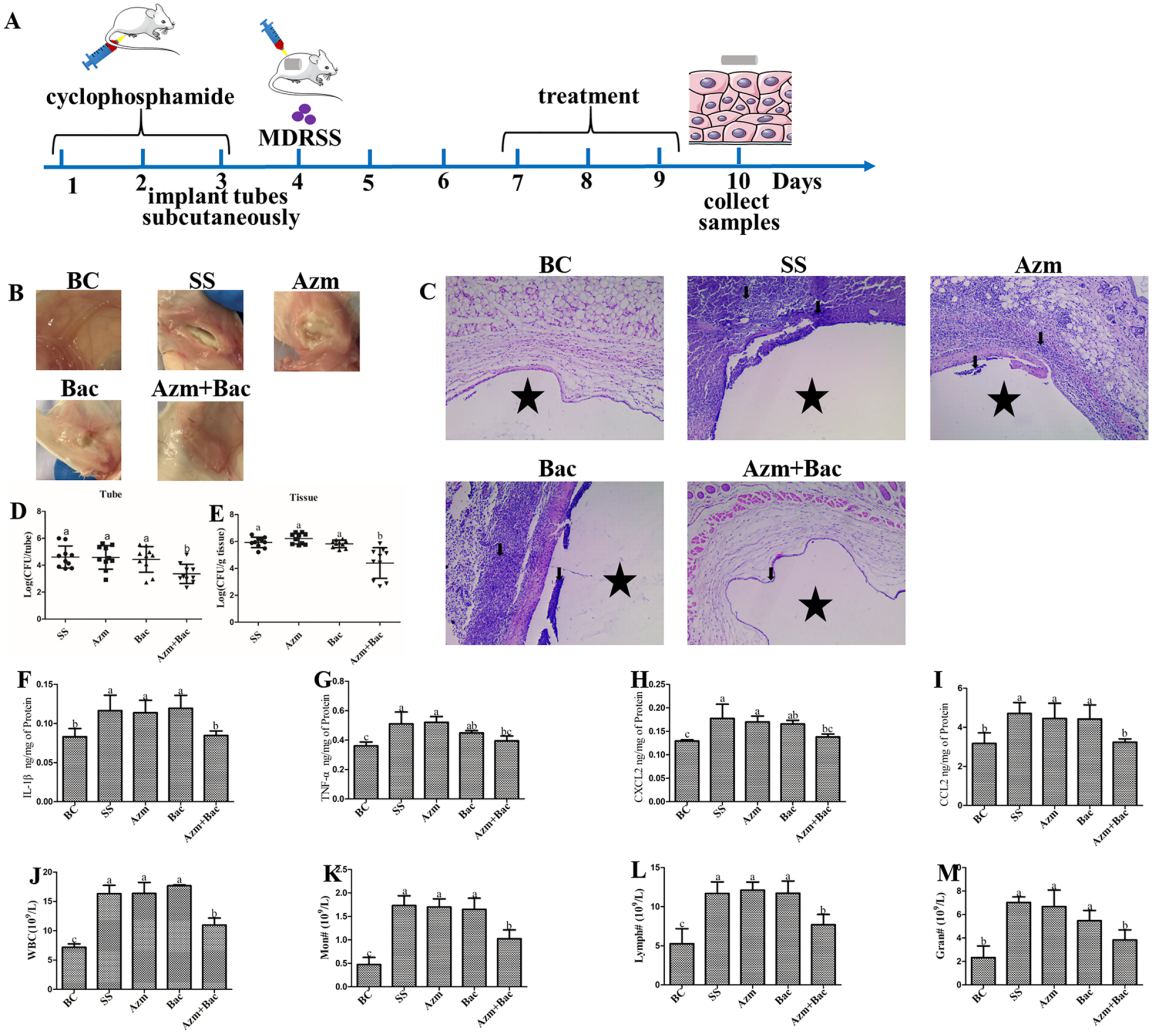
**The combination of azithromycin and baicalin is efficacious in vivo against MDRSS in an established murine implant infection model.** Azm: azithromycin-treated group; Bac: baicalin-treated group; Azm + Bac: the combination of azithromycin and baicalin-treated group. MDRSS: multidrug-resistant *Staphylococcus saprophyticus*; BC: blank control group; SS: multidrug-resistant *Staphylococcus saprophyticus* Liu-2016-Liyang, China-francolin (MDRSS) control group. Implanted subcutaneous tubes were infected with the MDRSS strain. Mice were sacrificed on the 10^th^ day. **A** Scheme of the experimental protocol for the murine implant infection model. **B** Histological changes. **C** Pathological changes were analysed by H&E staining. Asterisks indicate the position of the implant. The location of inflammatory cells is denoted by the arrows. Magnification: ×200. **D**, **E** MDRSS titers in ICR mouse implants and tissue around the implant. IL-1β (**F**), TNF-α (**G**), CXCL_2_ (**H**), and CCL_2_ (**I**) cytokine expression profiles for the tissue surrounding the implants infected with MDRSS were determined using ELISA kits. White blood cells (**J**), monocytes (**K**), lymphocytes (**L**), and neutrophils (**M**) in the blood were detected. Data are presented as the means ± SDs. In the same index, the graph bars in a-c with different letters on top represent statistically significant results (*p* < 0.05), whereas the bars labelled with the same letter correspond to results with no statistically significant differences.

Given the attractive potentiation of azithromycin and baicalin, the in vivo efficacies of azithromycin and baicalin were evaluated in a mouse flank implant infection model. Except for the BC group, a marked abscess was observed around the implant in all groups. However, the combination of azithromycin and baicalin alleviated the injury compared with those of the SS, Azm, and Bac groups. Under a microscopic examination, no lesions were observed in the soft tissues surrounding the implant in the BC group (Figure 6B). However, the histopathology of the mice in the SS, Azm, and Bac groups showed that a few periportal inflammatory cells (indicated by arrows) infiltrated into the infection sites. With the addition of azithromycin and baicalin, the number of inflammatory cells decreased (Figure 6C). Furthermore, the combination of azithromycin and baicalin significantly reduced the quantities of bacteria in the implants and surrounding soft tissue, the expression profiles of IL-1β and CCL_2_ in the surrounding soft tissue, and the number of white blood cells, lymphocytes, monocytes, and neutrophils in blood compared with those of the SS, Azm and Bac groups (*p* < 0.05). However, statistical significance was not reached between the SS, Azm, and Bac groups. The levels of TNF-α and CXCL_2_ in the Bac group were similar to those in the Azm + Bac group (Figure 6D–M). Together, these findings demonstrated that baicalin strengthened the efficacy of azithromycin in the treatment of biofilm infection.

## Discussion

Biofilm-related infections are troublesome and expensive to treat [26]. This is due to the reduced metabolic activity of biofilm-embedded cells and the protection conferred by the EPS. Currently, there are few new antibiotic candidates available [27]. Recently, synergistic drug combinations have often been used as a novel multimodal therapy to treat multidrug-resistant bacterial infections [28]. Previously, we found a synergistic effect of azithromycin and baicalin against MDRSS in the planktonic form [3]. In this study, we found that baicalin acted as an adjuvant to enhance the ability of azithromycin against MDRSS in the biofilm form (Tables 2 and 3; Figure 2). Biofilm development stages exhibit the following major events: initial attachment, biofilm maturation, and dispersal [29]. In our previous investigation, we found that the MDRSS biofilm dispersed from 24 to 48 h [20]. In this study, the biomass was not decreased by the combination of azithromycin and baicalin from 24 to 48 h (Figure 1A). However, the combination of azithromycin and baicalin significantly inhibited the detachment of MDRSS (Figure 1B). These results suggest that baicalin potentiates the effect of azithromycin by inactivating MDRSS and inhibiting the dispersal of biofilm.

The biofilm matrix of staphylococci is a complex glue that encases all of the cells in the mature structure, and it builds up from proteins, polysaccharides, and eDNA [29]. The extracellular matrix of biofilms provides a safety barrier to bacteria against the host immune system and the penetration of antimicrobial agents, which makes biofilms inherently difficult to treat, thereby resulting in an important health care issue [30]. SEM analysis showed that baicalin alone or combined with azithromycin significantly decreased the extracellular matrix compared with that of the blank control and Azm group (Figure 3). These results indicate that baicalin can disrupt the extracellular matrix of biofilms. Interestingly, after the addition of azithromycin and baicalin, the cells exhibited a distorted shape, and AKP and β-GAL were found outside the cells. Simultaneously, baicalin was also found to increase the AKP content in the supernatant (Figs. 3 and 4). AKP is an intercellular enzyme located between the cell wall and cell membrane of bacteria. β-GAL exists in the interior of bacteria. When the permeability of the bacterial cell wall and cell membrane increases, AKP and β-GAL leak outside the bacteria into the extracellular space [31–33]. These results demonstrate that baicalin increases the antimicrobial activity of azithromycin and finally leads to damage to cell walls and cell membranes. Therefore, we presumed that baicalin increased the penetration of azithromycin by disrupting the biofilm and cell wall matrix, thereby enhancing the efficacy of azithromycin.

The metabolism of the cell wall is regulated by the Walk/R system [34]. *yycH* and *yycI* play an important role in controlling *WalK* activity, and their absence leads to abnormal regulation of the WalK/R system, with associated growth and cell wall defects. Compared with planktonically cultivated bacteria, biofilms show an altered growth rate, metabolism, and gene expression profile [35]. The reduced metabolic activity of biofilm-embedded bacterial cells is responsible for resistance against antimicrobial agents and the host immune system [30]. In *S. aureus*, the WalK/R system is thought to play an important role in membrane permeability [36]. In the RT‒PCR assay, baicalin alone or in combination with azithromycin increased the transcript levels of the *walK*, *walR*, *yycH*, and *yycI* genes compared with the that of the azithromycin and blank control groups (Figure 5). These results reveal that the metabolism and membrane permeability of MDRSS in biofilm form are increased by the addition of baicalin. This study implied that there was a strong correlation between the transcript levels of WalK/R system-associated genes and the bactericidal index (Table 4). We speculate that baicalin increases the metabolism of embedded cells in biofilms, thereby enhancing the bactericidal activities of azithromycin.

The therapeutic potential of the combination of azithromycin and baicalin was further investigated in a mouse cutaneous infection model. The tissue injuries were relieved, and the bacterial load in the implants and tissues surrounding the infected implants were decreased under combined therapy (Figure 6). However, a good effect was not observed for the groups that were treated with azithromycin and baicalin alone. The morphology of bacteria in implants was not observed because of technology limitations. We previously demonstrated that the matrix of MDRSS biofilms consists of eDNA and proteins [20]. It is well known that eDNA and proteins are recognized by the innate immune system via TLR2 and TLR9 [25]. Then, the immune cells are recruited into the infection site. However, only a few inflammatory cells infiltrated into the infection sites, and the cytokines of neutrophil and macrophage recruitment (CXCL_2_ and CCL_2_, respectively) and activation (TNF-α and IL-1β) and the inflammatory cells in blood also decreased after treatment with the combination of azithromycin and baicalin (Figure 6). We presume that the combination of azithromycin and baicalin disrupts the biofilm matrix and kills the bacteria embedded in the extracellular matrix; therefore, the inflammatory cells were decreased in the infection sites. In the SS group, there were more inflammatory cells, but the bacterial burden did not decrease. These results demonstrate that macrophages infiltrate into biofilm infection sites, and few cells can migrate into the biofilm. It has been proposed that staphylococci biofilms promote the differentiation of macrophages towards a profibrotic M2 phenotype [25]. M2 macrophages could not phagocytize bacteria in biofilm form. However, planktonic staphylococci could promote the M1 phenotype immune response. The combination of azithromycin and baicalin may disrupt the structure of biofilms and improve the effect of M1-type macrophages.

In conclusion, our results suggested that the bacteriostatic effects of azithromycin in sessile MDRSS were improved by the addition of baicalin. Baicalin enhanced the effect of azithromycin in hindering the detachment of biofilm, disrupting the cell membranes and walls by modulating the relative expression of WalK/R system-associated genes. The combined use of azithromycin and baicalin in the treatment of biofilm infections was beneficial. These results demonstrate that baicalin has the potential to be an effective antibiotic adjuvant to increase the effect of azithromycin against preformed biofilms. This study provides an effective and simple combined strategy for the treatment of biofilm-related infections.

## Abbreviations

MDRSS: multidrug-resistant *Staphylococcus saprophyticus*-Liu-2016-Liyang, China-francolin
WalK: cell wall metabolism sensor histidine kinase
WalR: two-component response regulator
yycI: two-component system regulatory protein
yycH: two-component system activity regulator
CXCL_2_: CXC chemokine ligand 14
CCL_2_: CC chemokine ligand 2
TNF-α: tumour necrosis factor-α
IL-1β: interleukin 1 beta
eDNA: extracellular deoxyribonucleic acid
TLR2: toll-like receptor 2
TLR9: toll-like receptor 9
SEM: scanning electron microscopy
ELISA: enzyme-linked immunosorbent assay
RT-PCR: real-time polymerase chain reaction
